# Norovirus P particle-based active Aβ immunotherapy elicits sufficient immunogenicity and improves cognitive capacity in a mouse model of Alzheimer’s disease

**DOI:** 10.1038/srep41041

**Published:** 2017-01-20

**Authors:** Lu Fu, Yingnan Li, Yue Hu, Yayuan Zheng, Bin Yu, Haihong Zhang, Jiaxin Wu, Hui Wu, Xianghui Yu, Wei Kong

**Affiliations:** 1 National Engineering Laboratory for AIDS Vaccine, School of Life Sciences, Jilin University, Changchun 130012, China; 2 Key Laboratory for Molecular Enzymology and Engineering, the Ministry of Education, School of Life Sciences, Jilin University, Changchun 130012, China

## Abstract

Disease-modifying immunotherapies focusing on reducing amyloid-beta (Aβ) deposition are the main treatment for Alzheimer’s disease (AD). However, none of the Aβ immunotherapies has produced clinically meaningful results to date. The main reason for this lack of efficacy is that the vaccine induces insufficiently high antibody titers, as it contains small B-cell epitope of Aβ to avoid Aβ42-specific T-cell activation. With the aim of generating a potent AD vaccine, we designed the protein PP-3copy-Aβ1-6-loop123, comprising three copies of Aβ1-6 inserted into three loops of a novel vaccine platform, the norovirus P particle, which could present Aβ at its surface and remarkably enhance the immunogenicity of the vaccine. We demonstrated that PP-3copy-Aβ1-6-loop123 was able to elicit high antibody titers against Aβ42, without causing T-cell activation, in AD mice regardless of their age. Importantly, PP-3copy-Aβ1-6-loop123 treatment successfully reduced amyloid deposition, rescued memory loss, and repaired hippocampus damage in AD mice. The Aβ antibodies induced by this active immunotherapy reacted with and disrupted aggregated Aβ, reducing its cellular toxicity. In addition, our results suggested PP-3copy-Aβ1-6-loop123 immunization could restore Aβ42 homeostasis in both the serum and brain. Thus, the P particle-based Aβ epitope vaccine is a sufficiently immunogenic and safe immunotherapeutic intervention for Alzheimer’s disease.

Alzheimer’s disease (AD) is a progressive age-related neurodegenerative disorder that affects more than 46 million people worldwide^1^. As the etiology and pathophysiology of AD are multifactorial and complex, only a few symptomatic treatments, such as cholinesterase inhibitors and memantine, are approved for AD therapy; however, no disease-modifying therapies are currently available^2^,^3^,^4^. The amyloid cascade hypothesis posits that the deposition of amyloid β (Aβ) in the brain is the central pathological hallmark of AD^5^,^6^,^7^,^8^. Thus, over the past 15 years, various active and passive Aβ immunotherapies have progressed from preclinical studies in AD mouse models to clinical trials in humans, suggesting that the enhancement of Aβ clearance may be the most promising therapeutic options for AD^9^,^10^. Unfortunately, until now, no data have been reported regarding the most potent Aβ immunotherapy, which is currently in phase III clinical trials^9^,^11^,^12^,^13^.

The first clinical trial of the active AD vaccine AN1792, which used full-length Aβ42 formulated in the adjuvant QS21, was halted at phase II when 6% of the trial subjects developed aseptic meningoencephalitis^14^. Further studies of affected patients demonstrated that a strong Aβ-reactive T-cell autoimmune response had occurred due to the use of full-length Aβ42, which contains T-cell epitopes residing in amino acids 15 to 42^15^. Thereafter, many groups developed Aβ-based epitope vaccines composed of different N-terminal regions of Aβ42, ending between amino acids 6 and 15 to avoid strong T-cell responses^16^,^17^,^18^,^19^,^20^. Recently, a preclinical study of second-generation active Aβ immunotherapy CAD106, used Aβ1-6 as an epitope coupled to a virus-like particle Qβ and demonstrated that the vaccine induced efficacious Aβ antibody titers without T-cell responses in amyloid precursor protein (APP) transgenic mice^21^. Thus, Aβ1-6 is a safe immunogen, and anti-Aβ antibodies generated following inoculation of vaccine containing the Aβ1-6 epitope might counteract the adverse effects of synthetic Aβ *in vitro*.

Aside from the safety issue of T-cell-mediated autoimmune responses, another problem with the failed Aβ immunotherapies was the modest immunogenicity. In the course of avoiding Aβ42-specific T-cell activation, the second-generation Aβ active vaccines were all produced with a small B-cell epitope of Aβ, which has low immunogenicity. To compensate for this low immunogenicity, several AD active immunotherapies applied different vaccine carriers to provide Th cell epitopes, which can stimulate B cells to produce maximal antibody titers^19^. For instance, CAD106 used phage Qβ as the vaccine platform and the ACC001 vaccine used diphtheria toxin to carry the Aβ1-7 epitope^9^,^21^. However, none of the above AD vaccines has produced clinically meaningful results, indicating that a more optimal and potent vaccine platform is required for the future development of Aβ epitope vaccines.

Noroviruses (NoVs), formally called Norwalk-like viruses, are associated with human epidemic acute gastroenteritis^22^. The NoV contains a protein capsid protein (VP1), which has two major domains, the shell (S) domain and the protruding (P) domain^23^. The P particle (PP) is a subviral nanoparticle formed by 24 copies of the P domain, which is easily produced, extremely stable and highly immunogenic^24^. The structural analysis of the P domain revealed three surface loops on its distal surface, which have been shown to be the sites of foreign epitope insertion and presentation^25^. Thus, the PP is considered to be an excellent multipurpose platform for antibody induction and vaccine development against many pathogens, including rotavirus and influenza virus^26^,^27^,^28^.

With the aim of generating a potent AD vaccine that elicits sufficiently high immunogenicity and efficiently improves cognitive capacity, we designed the protein PP-3copy-Aβ1-6-loop123, comprising three copies of Aβ1-6 inserted into all three loops of the norovirus P particle. PP-3copy-Aβ1-6-loop123 was able to elicit high antibody titers against Aβ42, without causing T-cell activation, in AD mice. Most importantly, PP-3copy-Aβ1-6-loop123 treatment successfully reduced amyloid deposition and rescued memory loss in AD mice. In addition, our results indicated that PP-3copy-Aβ1-6-loop123 immunization could restore Aβ homeostasis in the brain via both the “direct-targeting” and “peripheral sink” pathways^29^.

## Results

### The development and characterization of PP-3copy-Aβ1-6-loop123

**Figure 1:**
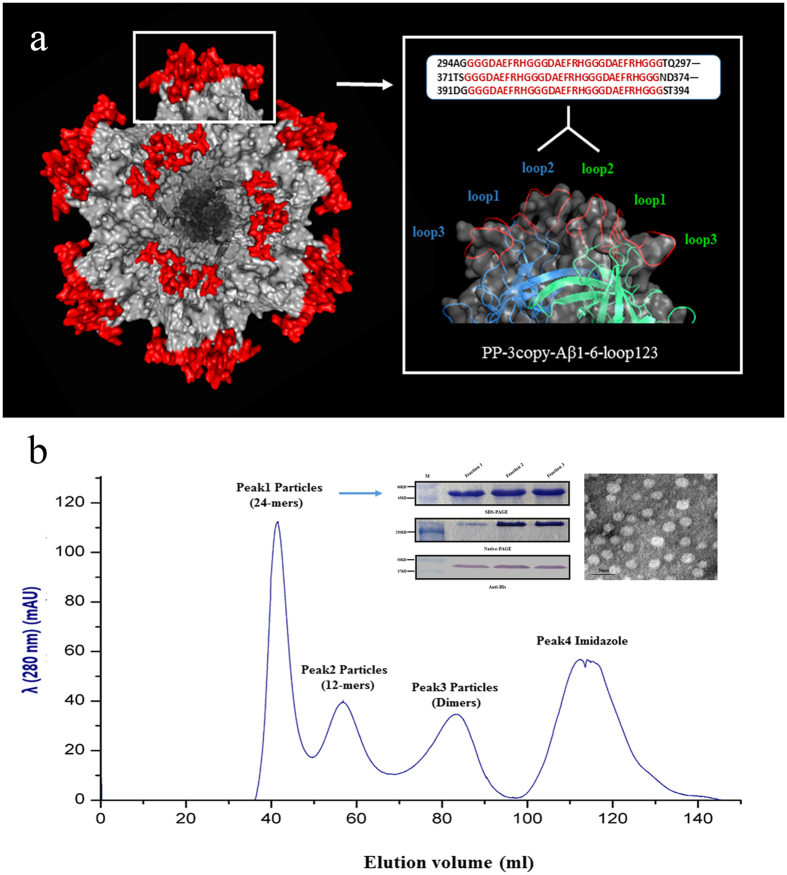
The construction and purification of PP-3copy-Aβ1-6-loop123. (**a**) Prediction of the 24-mers structure of PP-3copy-Aβ1-6-loop123 and homology remodeling of the dimer form of PP-3copy-Aβ1-6-loop123. Red part represented three copies of Aβ1-6, and white part represented P particle backbone. (**b**) Size exclusion chromatography of PP-3copy-Aβ1-6-loop123 using a Superdex^TM^ 200 column. Recombinant P particles from peak 1 were analyzed by the following methods: SDS-PAGE, native-PAGE, anti-His western blot, and TEM analysis. The scale bar of TEM observations is 50 nm.

To produce maximal antibody titers and avoid the activation of autoreactive T-cells, we chose Aβ1-6 as the antigen and the NoV P particle as the vaccine platform to develop a novel AD protein vaccine. In our previous study, we successfully constructed and purified five recombinant P particle vaccines containing different copies of Aβ1-6 and distinct loop insertion forms^30^. The results showed that among the five AD protein vaccines, PP-3copy-Aβ1-6-loop123 was the most effective vaccine for inducing Aβ antibodies *in vivo*. In PP-3copy-Aβ1-6-loop123, an epitope containing three copies of Aβ1-6 was inserted into loop 1, loop 2, and loop 3 of the P domain, which enabled all the Aβ1-6 antigens to be presented on the surface of the recombinant P particle (Fig. 1a).

Following initial purification by Ni-NTA, we further purified PP-3copy-Aβ1-6-loop123 using a Superdex^TM^ 200 column to separate the P particles that had formed a 24-mer, which might be more highly immunogenic (Fig. 1b and S1). The SDS-PAGE and Western blot analysis showed that the molecular weight of the 24-mer P particle in peak 1 was about 45 kDa. Native-PAGE analysis suggested that the recombinant P particle could still form the multimer *in vitro*. In addition, TEM observation suggested that the size of PP-3copy-Aβ1-6-loop123 was about 20 nm, that these recombinant P particles were evenly distributed and that the particles appeared to be globular.

### The PP-3copy-Aβ1-6-loop123 protein vaccine was able to induce high Aβ42 antibody titers, whilst avoiding Aβ-specific T-cell activation, in wild-type mice

**Figure 2:**
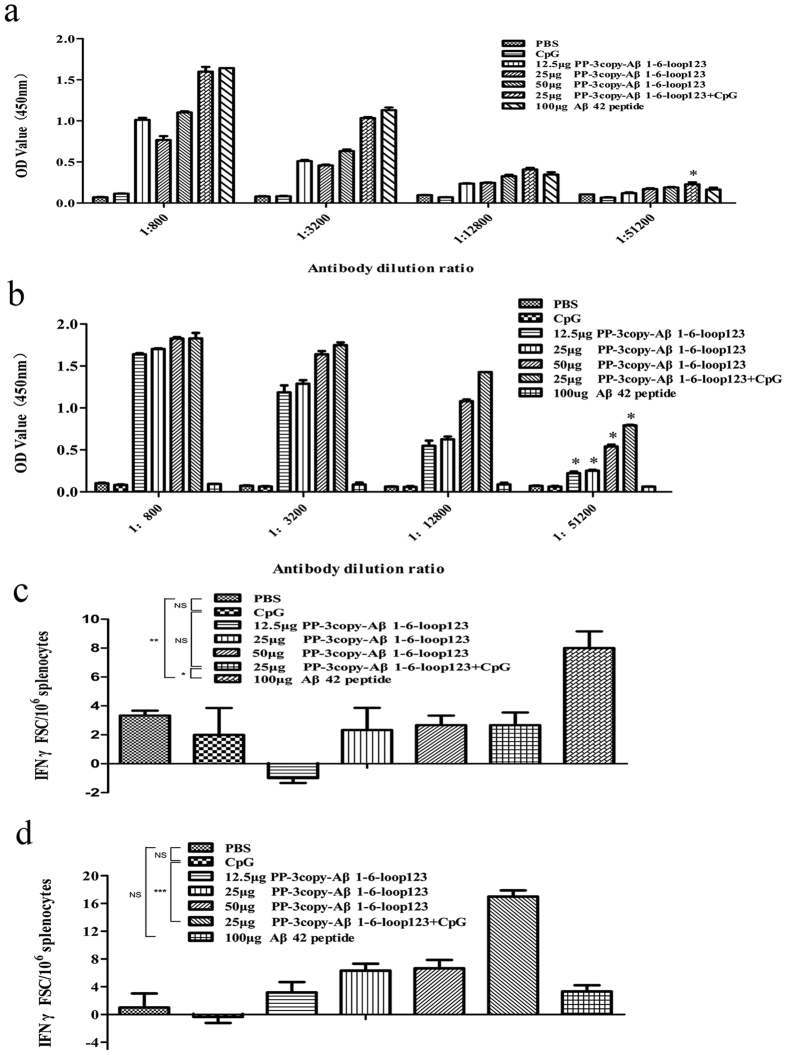
The immunogenicity and safety evaluation of PP-3copy-Aβ1-6-loop123 in WT C57BL/6 mice. (**a**) Dose-response study of PP-3copy-Aβ1-6-loop123 in C57BL/6 mice. Aβ42 antibody levels in the serum of mice after four immunizations with PBS, CpG, PP-3copy-Aβ1-6-loop123 in different dosage or Aβ42 were examined by ELISA. Absorbance values greater than twofold over the background were considered positive and are marked with *. (**b**) Detection of antibody levels against P particle in different mice groups after four immunizations with PP-3copy-Aβ1-6-loop123. Absorbance values greater than twofold over the background were considered positive and are marked with *. (**c**) Activation of Aβ42-specific T-cells from each group were assessed by Elispot assay (T-cell activation). Isolated spleen cells from each group were stimulated with Aβ42 peptide and IFN-γ-secreting T-cell spots were quantified. Statistically significant difference were observed between PBS and Aβ42 peptide immunized mice (P = 0.0375), as well as 25 μg PP-3copy-Aβ1-6-loop123 + CpG and Aβ42 peptide immunized groups (P = 0.0472). *p < 0.05, NS = non-significant. (**d**) The determination of the stimulation of P particle-specific T-cells of each group by Elispot assay (T-cell activation). Isolated spleen cells from each group were stimulated with P particle protein and IFN-γ-secreting T-cell spots were quantified. Statistically significant differences were observed between CpG and 25 μg PP-3copy-Aβ1-6-loop123 + CpG immunized groups (P = 0.0004). ***p < 0.0001, NS = non-significant. All the results were expressed as mean values ± SEM. ANOVA test was used to analyze the statistical significance of the data.

We first investigated the immunogenicity of the PP-3copy-Aβ1-6-loop123 protein vaccine in wild-type mice. In contrast to the PBS group, a high titer of Aβ42-specific antibody was detected in the PP-3copy-Aβ1-6-loop123-immunized mice (Fig. 2a). We also measured the antibody titers against the P particle elicited by PP-3copy-Aβ1-6-loop123. As shown in Fig. 2b, PP-3copy-Aβ1-6-loop123 also elicited a high titer of P particle-specific antibody, confirming that this recombinant P particle is immunogenic. The dose-response study indicated that the optimal immunization method was 25 μg PP-3copy-Aβ1-6-loop123 formulated with CpG and this method was therefore chosen for further studies in APP/PS1 transgenic mice (Fig. 2a).

To confirm that PP-3copy-Aβ1-6-loop123 is a safe active immunotherapy for AD without inducing T-cell responses, we further assessed the activation of Aβ-specific T- cells when mice were sacrificed 15 days after the final immunization. As expected, vaccination with the PP-3copy-Aβ1-6-loop123 protein vaccine in any dose showed background values when stimulated with peptide Aβ1-42, confirming that Aβ1-6 peptide lacks the T-cell epitope (Fig. 2c). We also found that the major antibody isotype present in the serum of mice treated with PP-3copy-Aβ1-6-loop123 was IgG2b, which is stimulated during Th2-type immune responses (data not shown). In comparison, Aβ42 peptide immunization stimulated a threefold increase in the number of Aβ-specific T-cells after stimulation with Aβ42. In addition, immunization with different doses of PP-3copy-Aβ1-6-loop123 all resulted in a strong stimulation of P particle-specific T-cells, which provide the additional T-cell mediated help required by anti-Aβ specific B cells (Fig. 2d).

### The antibody induced by PP-3copy-Aβ1-6-loop123 reduces the formation rate of Aβ aggregates and blocks the toxicity of Aβ oligomers in cells

**Figure 3:**
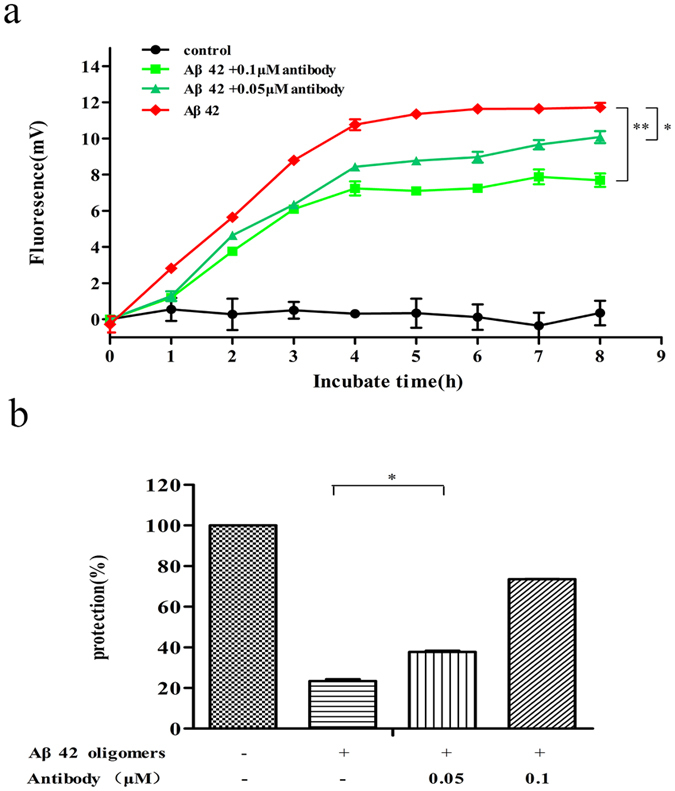
The *in vitro* function of antibodies induced by PP-3copy-Aβ1-6-loop123. The results are expressed as mean values ± SEM. (**a**) The Aβ aggregation inhibition assay. The serum antibodies from PP-3copy-Aβ1-6-loop123 vaccine immunized mice remarkably inhibited Aβ aggregation. *p = 0.0161, **p = 0.0014. (**b**) Aβ42 oligomer toxicity assay in PC12 cells. The antibodies induced by PP-3copy-Aβ1-6-loop123 treatment reversed the oligomer-induced toxicity in a concentration-dependent manner, *p = 0.0203.

To further examine whether the stimulated Aβ antibodies were functional *in vitro*, Aβ42 antibodies induced by PP-3copy-Aβ1-6-loop123 in wild-type mice were purified and tested for their ability to inhibit Aβ aggregate formation. As shown in Fig. 3a, the antibody efficiently inhibited the aggregation of Aβ in a dose-dependent manner and remarkably reduced the formation rate of Aβ aggregates. The Aβ antibody could also efficiently degrade Aβ oligomers *in vitro* (Fig. S2). In addition, the antibodies were assessed for their ability to block the toxicity of Aβ oligomers in cells. The results showed that those antibodies efficiently blocked Aβ42 oligomer-induced toxicity to PC12 cells in a concentration-dependent manner (Fig. 3b). When 0.1 μM of the purified Aβ antibodies induced by PP-3copy-Aβ1-6-loop123 was applied to the cells, the level of protection reached 80% compared to the blank control, indicating that the PP-3copy-Aβ1-6-loop123 protein vaccine could stimulate functional Aβ antibodies *in vivo*.

### The PP-3copy-Aβ1-6-loop123 protein vaccine is sufficiently immunogenic in an AD mouse model

**Figure 4:**
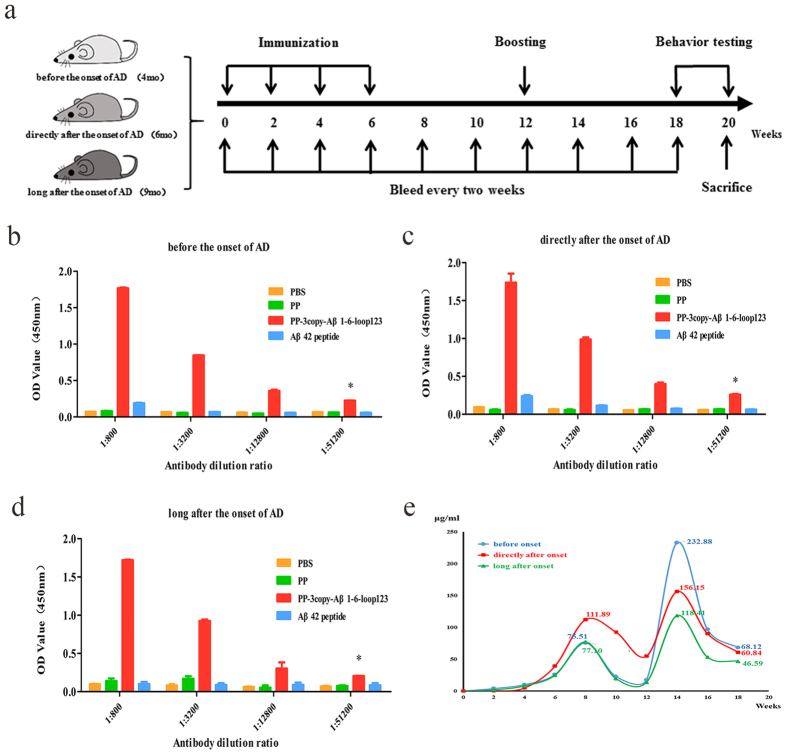
The determination of Aβ42-specific antibody titers in APP/PS1 transgenic mice after PP-3copy-Aβ1-6-loop123 immunization. Control groups received PBS, PP protein or Aβ42 peptide. The results are expressed as mean values ± SEM. (**a**) Grouping and immunization strategy for APP/PS1 transgenic mice. Aβ42 antibody titers after the forth immunization of PP-3copy-Aβ1-6-loop123 are detected in cohorts treated before the onset of AD (**b**), directly after the onset of AD (**c**), and long after the onset of AD (**d**). Absorbance values greater than twofold over the background were considered positive and are marked with *. (**e**) A comparison of average antibody titers and durations in the three age cohorts of mice immunized with PP-3copy-Aβ1-6-loop123.

Next, we investigated the immunogenicity of PP-3copy-Aβ1-6-loop123 in APP/PS1 transgenic mice. Three cohorts of transgenic mice were immunized with PP-3copy-Aβ1-6-loop123 following the prime-boost strategy (Fig. 4a). APP/PS1 transgenic mice were divided into three cohorts. One cohort was treated before the onset of AD at 4 months, and the other two cohorts were immunized directly after the onset of AD at 6 months, or long after the onset of AD at 9 months. After the fourth immunization, PP-3copy-Aβ1-6-loop123 successfully induced a strong and specific antibody response against Aβ42 in all the cohorts of transgenic mice (Fig. 4b–d). In contrast, no Aβ antibody response was detected in the control group mice. Median antibody titers in the vaccine-immunized mice were in the range of 70–110 μg/ml after the fourth injection; the mice treated directly after the onset of plaque formation generated the highest Aβ antibody titers among the three cohorts (Fig. 4e). Following the boosting immunization, the Aβ antibody concentrations were significantly enhanced, especially for the mice treated before the onset of AD. Finally, the Aβ antibody titer was maintained at a high level of around 50 μg/ml, providing continuous treatment for the AD mice (Fig. 4e). However, to our surprise, Aβ42 peptide-immunized transgenic mice did not develop a high Aβ-specific immune response. The Aβ antibody titer induced by PP-3copy-Aβ1-6-loop123 in APP/PS1 transgenic mice was also remarkably lower than that of WT mice. This unexpected result might be explained by the weak immune response of APP/PS1 transgenic mice.

### PP-3copy-Aβ1-6-loop123 efficiently improves cognitive capacity and repairs damage to the hippocampus in APP/PS1 transgenic mice

**Figure 5:**
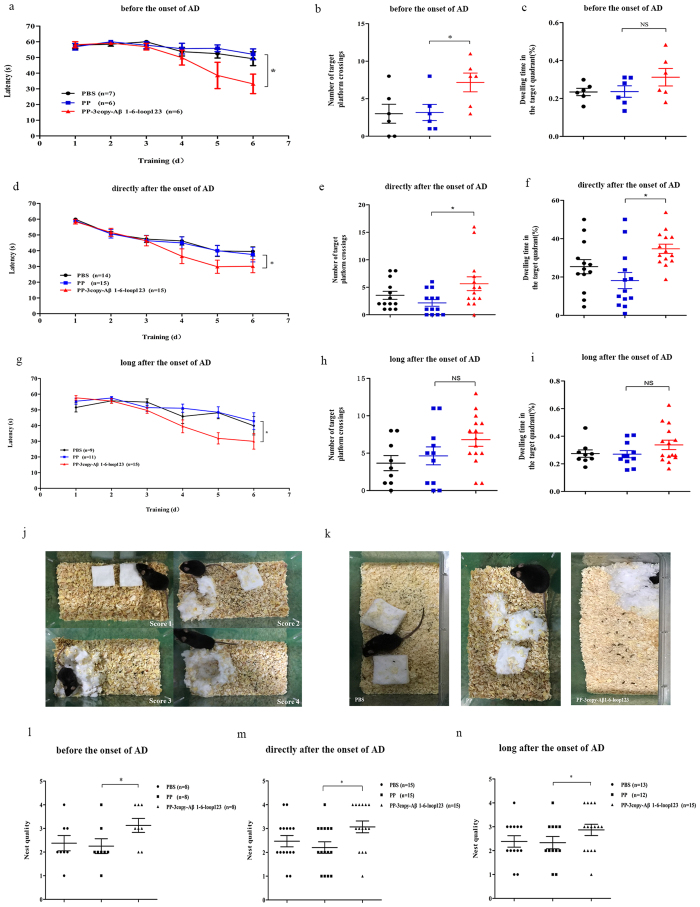
A behavioral analysis of the PP-3copy-Aβ1-6-loop123 immunized APP/PS1 transgenic mice. (**a**–**i**) Represent the Morris water maze test. For the cohorts treated before the onset of AD, the latencies are shown in (**a**) (*p* = 0.0122), total numbers of target platform crossings are shown in (**b**) (*p* = 0.0205), and the time spent by animals in the target quadrant are shown in (**c**). (**a,b** and **c**) Share the same set of tags. For the cohorts treated directly after the onset of AD, the latencies are shown in (**d**) (*p* = 0.0396), total numbers of target platform crossings are shown in (**e**) (*p* = 0.0409), and the time spent by animals in the target quadrant are shown in (**f**) (*p* = 0.0221). (**d**,**e** and **f**) share the same set of tags. For the cohorts treated long after the onset of AD, the latencies are shown in (**g**) (*p* = 0.0476), total numbers of target platform crossings are shown in (**h**), and the time spent by animals in the target quadrant are shown in (**i**). (**g**,**h** and **i**) Share the same set of tags. (**j**–**n**) Relate to the nest-building test. (**j**) Standard for evaluation. (**k**) Representative results of the nest-building test from the cohort immunized directly after the onset of AD. The quantitative analyze results from the cohort treated before the onset of AD (**l**) (*p* = 0.0441), directly after the onset of AD (**m**) (*p* = 0.0306), and long after the onset of AD (**n**) (*p* = 0.0417). All the results are expressed as mean values ± SEM. Number of animals per group is indicated in the bracket. ANOVA test was used to analyze the statistical significance of the data. *p < 0.05, NS = non-significant.

To determine if the vaccination using PP-3copy-Aβ1-6-loop123 had beneficial functional consequences in the APP/PS1 transgenic mice, we assessed the spatial learning and memory capacity of the immunized transgenic mice using a Morris water maze. When we assessed the escape latency of a random search for the hidden platform during the pre-training test, all mice immunized with the PP-3copy-Aβ1-6-loop123 vaccine in the three age cohorts performed significantly better than mice from the control group (*p* < 0.05) (Fig. 5a,d and g). After removing the hidden platform, PP-3copy-Aβ1-6-loop123-treated mice still concentrated on searching for the platform in the quadrant where it had previously been located, and performed better with regard to passing over the platform site (*p* < 0.05) (Fig. 5b,e and h). On the contrary, immunization with PBS or PP produced no improvement in the performance of transgenic mice in the Morris water maze test (*p* > 0.05). We also found that the vaccine-immunized mice had an increased dwelling time in the target quadrant compared to PBS-immunized mice (Fig. 5e,f and i). These results indicated that PP-3copy-Aβ1-6-loop123 immunotherapy improved the cognitive capacity of APP/PS1 transgenic mice. Moreover, across the three age cohorts, mice treated directly after the onset of AD (6 months) exhibited a significantly better improvement compared with mice treated long after the onset (9 months), suggesting that early intervention during the course of disease may have a better effect than later in disease.

Non-maternal nest building performance is sensitive to hippocampus damage and is used to evaluate murine models of psychiatric disorders^31^,^32^,^33^,^34^. We therefore compared the nest building capacity of AD mice immunized with PBS, PP or PP-3copy-Aβ1-6-loop123. The standard for assessing nest building performance is shown in Fig. 5j. Control studies indicated that APP/PS1 transgenic mice injected with PBS or PP were not able to build their nest well: the median score was only two (Fig. 5l–n). In contrast, immunization with PP-3copy-Aβ1-6-loop123 resulted in an enhanced nest building capacity in all the three cohorts, indicating that the PP-3copy-Aβ1-6-loop123 vaccine could repair damage to the hippocampus and restore the nest-building capacity of AD mice. Representative results of the nest-building test from the cohort immunized with PP-3copy-Aβ1-6-loop123 directly after the onset of AD are shown in Fig. 5k. Consistent with the results from Morris water maze test, mice treated before or directly after the onset of AD exhibited a more significant improvement in nest building ability compared to the cohort immunized long after the onset of AD, suggesting that this P particle-based Aβ epitope vaccine could be more effective if it is initiated before or at the early stages of AD.

### Amyloid deposition in AD mice is reduced following PP-3copy-Aβ1-6-loop123 immunization

**Figure 6:**
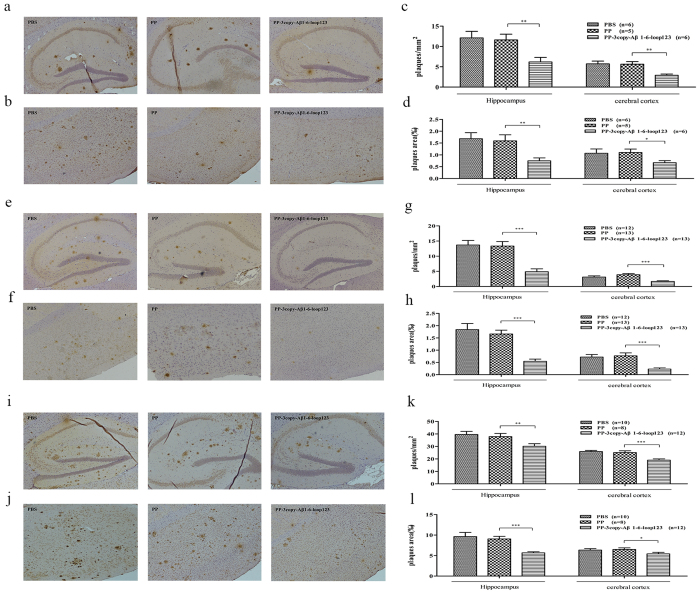
The immunohistochemical analysis of amyloid plaque deposits in the cortical and hippocampal sections of APP/PS1 transgenic mice after treatment with PP-3copy-Aβ1-6-loop123, PBS or PP protein. Representative immunohistochemical photomicrographs of amyloid plaques in the hippocampus and cerebral cortex of the cohorts treated before, directly after, and long after the onset of AD are given in (**a,e,i** and **b,f,j**), respectively. The number of plaques in both the hippocampus and cerebral cortex are shown for the cohorts treated before the onset of AD (**c**), directly after the onset of AD (**g**), and long after the onset of AD (**k**). The area ratio of plaques are shown for cohorts treated before the onset of AD (**d**), directly after the onset of AD (**h**), and long after the onset of AD (**l**). The results are expressed as mean values ± SEM. The statistical significance of the data was analyzed by ANOVA test. Number of animals per group is indicated in the bracket. *p < 0.05, **p < 0.01, ***p < 0.001.

We first measured the reduction in amyloid deposition following PP-3copy-Aβ1-6-loop123 immunization in before the onset of AD group. At the end of this study (9 months), only a few plaques could be observed in the hippocampus and cerebral cortex of the PP-3copy-Aβ1-6-loop123-treated mice compared to the control group (Fig. 6a and b). In addition, the number of plaques in the hippocampus and cerebral cortex was reduced by 48 and 49%, respectively, compared with the PP-treated group, and the relative area covered by plaques was reduced by 55 and 37% (Fig. 6c,d and Table 1).

In the second study, which involved a therapeutic mode of treatment, we treated AD mice directly after the onset of AD from 6 to 11 months. PP-3copy-Aβ1-6-loop123 significantly reduced the deposition of amyloid plaques in AD mice, whereas no effect was found in the mice immunized with PP- and PBS (Fig. 6e and f). The reductions in plaque number and plaque area were 65 and 70%, respectively, in the hippocampus and 46 and 67% in the cerebral cortex, compared to the PP group (Fig. 6g and h and Table 1).

In a further study, elderly mice were treated from 9 to 14 months. Treatment started long after the onset of AD, by which point the mice already carried a high amyloid load. The results showed that the PP-3copy-Aβ1-6-loop123 immunization was still effective, although the reduction in plaque load following vaccination was slightly lower compared with the other two cohorts (Fig. 6i and j). Nevertheless, there was a remarkable reduction in plaque load in both the hippocampus (number, 23%; area, 41%) and cerebral cortex (number, 26%; area, 14%) (Fig. 6k and l and Table 1).

Amyloid plaque deposits in the cortex and hippocampus of APP/PS1 transgenic mice increased with age. However, PP-3copy-Aβ1-6-loop123 treatment could significantly reduce the amyloid load compared with the age-matched control group. Moreover, the results suggested that the treatment would be more effective when initiated at the early stages of AD.

### PP-3copy-Aβ1-6-loop123 immunization restores Aβ42 homeostasis in the serum and brain *in vivo*

**Figure 7:**
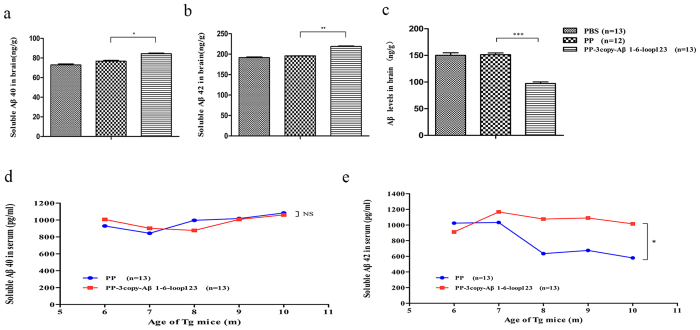
The determination of biomarker levels in PP-3copy-Aβ1-6-loop123, PBS or PP immunized APP/PS1 transgenic mice treated directly after the onset of AD. The levels of soluble Aβ40 (**a**) (*p* = 0.0164), soluble Aβ42 (**b**) (*p* = 0.0087) and aggregated Aβ (**c**) (*p* = 0.0007) in the brain of mice after treatment. (**a,b,** and **c**) share the same set of tags. (**d**) Soluble Aβ40 level in the serum of the groups immunized with PP and PP-3copy-Aβ1-6-loop123 or PP. (**e**) Level of soluble Aβ42 in the serum after immunization. Statistically significant differences were observed between PP and PP-3copy-Aβ1-6-loop123 immunized groups (*p* = 0.0377). The results are expressed as mean values ± SEM. Number of animals per group is indicated in the bracket. *p < 0.05, **p < 0.01, ***p < 0.001.

The levels of Aβ40 and Aβ42 in the serum and brain were measured in groups of mice treated directly after the onset of AD. The levels of soluble Aβ40 and Aβ42 were increased, and levels of Aβ were significantly decreased, in the brains of the PP-3copy-Aβ1-6-loop123-immunized group compared to the control group (Fig. 7a–c). Meanwhile, the Aβ42 concentration in the serum of the control group declined slowly with age but this phenomenon was not observed in PP-3copy-Aβ1-6-loop123-treated mice (Fig. 7e). The change in serum levels of Aβ40 were the same in the vaccine and control groups (Fig. 7d). Thus, PP-3copy-Aβ1-6-loop123 immunization could directly target Aβ aggregates in the brain and restore Aβ42 homeostasis in both the serum and brain.

### Proinflammatory cytokine analysis of the PP-3copy-Aβ1-6-loop123 vaccine in transgenic mice

Safety is a very important issue in the preclinical research of AD vaccines, so we checked whether PP-3copy-Aβ1-6-loop123 induced inflammatory reactions in APP/PS1 transgenic mice. Levels of the proinflammatory cytokines IL-2, IL-4, IL-10, and IFN-γ in the brain in all three groups were below the detection limit. Levels of the proinflammatory cytokines IL-6, IL-1β, and TNF-α were significantly increased in AD mice compared with WT mice, confirming that inflammation is an important characteristic of Alzheimer’s disease. We also observed a slight but non-significant decrease in the concentrations of IL-6, IL-17A, IL-1β, and TNF-α in PP-3copy-Aβ1-6-loop123 immunized mice compared with the PBS group, indicating proinflammatory cytokines decreased after treatment with PP-3copy-Aβ1-6-loop123 (Fig. S3).

## Discussion

The pathophysiology of AD involves disturbances and imbalances occurring by a variety of mechanisms indicating a complicated disease process. No disease-modifying therapies are currently available for AD. Multiple lines of evidence suggest that the deposition of Aβ, along with the slowing of Aβ clearance, are central to the onset and progression of AD^5^,^7^,^8^. Unfortunately, most Aβ-targeted therapeutics have failed^35^. Reasons for the previous failures of anti-Aβ drugs include choosing an inappropriate target, an incomplete understanding of the drug’s pharmacokinetics or inappropriate dosage and treatment time^35^. Unlike anti-Aβ drug therapy, active immunotherapy relies on the patient’s own immune system to induce a polyclonal response with antibodies that differ with respect to their binding affinity for a number of toxic Aβ species. Furthermore, active immunotherapy can produce persistent levels of Aβ antibody titers with less-frequent administration^9^. Taking these factors into account, immunotherapy maybe the most promising therapy for AD. Unfortunately, most of the active or passive Aβ vaccines focusing on reducing amyloid deposition in the brain showed great initial potential but subsequently failed in clinical trials^36^,^37^,^38^. However, the long-term functional benefits of AN1792 were reported^39^ and the early results from a BIIB037 phase I trial and studies of aducanumab which removes amyloid plaques from the brain and slows progression of the disease appear promising^40^. Therefore, this recent trial provides strong support for the ongoing use of Aβ as a therapeutic target. In this study, we characterized a P particle-based AD protein vaccine, PP-3copy-Aβ1-6-loop123, which is an active Aβ epitope vaccine designed to elicit sufficiently high immunogenicity and to efficiently improve cognitive capacity. We demonstrated that PP-3copy-Aβ1-6-loop123 was able to elicit high Aβ antibody titers, reduce amyloid deposition, and improve cognitive capacity in an AD mouse model.

All mice immunized with PP-3copy-Aβ1-6-loop123 developed high Aβ antibody titers, regardless of their age. The induced Aβ antibody had a high affinity to Aβ oligomers and fibers, and could effectively inhibit Aβ aggregation *in vitro* and neutralize the toxicity induced by Aβ oligomers in a cellular assay. This finding is significant because this AD vaccine contains a small B-cell epitope of Aβ that has low immunogenicity. P particle-based vaccine-immunized mice also induced high levels of anti-P particle antibody. Most importantly, P particle-specific T-cells were activated in treated mice, indicating that the P particle offers specific foreign T-cell epitopes and triggers the provision of T-cell help to the Aβ-specific B cells. We have also confirmed that immunization with the P particle-based Aβ vaccine did not induce Aβ42-specific T-cell activation. Thus, the P particle successfully enhanced the immunogenicity of Aβ1-6 antigen whilst minimizing potential side effects.

The lifespan of the induced antibody in peripheral blood is an important factor in the efficacy of AD immunotherapy. In the PP-3copy-Aβ1-6-loop123-immunized group, the mean antibody titers were in the 70–110 μg/ml range during the prime immunization, which is slightly stronger than the antibody titers elicited by the Qβ-based Aβ epitope vaccine CAD106 that has already progressed into human clinical trials^21^. Subsequently, the antibody titers slowly declined to 20–60 μg/ml within four weeks. After the boost immunization, the antibody titers increased approximately twofold, compared with the titers following the prime immunization, and then dropped to 50–70 μg/ml within one month. Thus, the prolonged duration in the blood of the Aβ antibody during treatment with PP-3copy-Aβ1-6-loop123 produced functional improvements, such as amyloid deposition clearance and retard of memory loss, in an AD mouse model.

We also compared the readouts for amyloid load reduction in the three immunization cohorts of different ages. The mice immunized directly after the onset of AD had higher Aβ antibody titers than mice treated long after the onset of AD, in both prime and boost immunization. As a result, the reduction in amyloid deposition in the mice injected directly after the onset of AD was significantly larger than the reduction in mice treated long after the onset of AD. In addition, a greater reduction in amyloid deposition was found when mice were immunized before the onset of AD as opposed to immunization long after the onset of AD, indicating that immunotherapy is more efficacious when used in the early stage of the AD, especially in the prodromal stage. Thus, our studies indicated a correlation between Aβ antibody titers and the effects on amyloid deposition. We also noticed that the Aβ reduction in mice treated before the onset of AD was not greater than in mice immunized directly after the onset of AD, although induced Aβ antibody titers were greater in the former group than in the latter group following the boost immunization. We deduce from this observation that the antibody titers following the prime immunization directly determine the functional outcome, as mice immunized directly after the onset of AD generated the highest Aβ antibody titers among the three age cohorts and the extent of amyloid accumulation clearance was the greatest in these mice. As the pathophysiology of AD is complex and the neurological damage is irreversible, it is difficult to reverse the effects of the disease once it has begun to develop Therefore, therapeutic trials carried out early during the course of the disease may have better effect.

The efficacy of the P particle-based Aβ epitope vaccine on the restoration of cognitive function in APP/PS1 transgenic mice was also investigated. In the Morris water maze experiment, during the six training days, PP-3copy-Aβ1-6-loop123-immunized mice in all three age cohorts remembered the location of the platform and exhibited the shortest latency. After removing the platform, the vaccine-immunized mice were able to accurately find the prior location of the platform whereas mice immunized with either PBS or the P particle did not. These results demonstrated that PP-3copy-Aβ1-6-loop123 immunization effectively improved the spatial learning ability and rescued memory loss in AD mice, regardless of their age. Similar results were obtained in the nest-building test: the mean score of the Aβ epitope vaccine-immunized mice was significantly higher than the scores of the other groups, providing evidence for the restoration of hippocampus function in the vaccine-treated mice.

Several mechanisms could explain the antibody-mediated clearance of the Aβ load *in vivo*. The direct-targeting mechanism proposes that a small amount of serum Aβ antibodies can cross the blood-brain barrier (BBB), bind with the Aβ aggregates, and then induce the phagocytosis of the antigen-antibody complexes via the Fc portion of the antibody. In an alternative mechanism known as the “peripheral sink” pathway, anti-Aβ antibodies in the peripheral blood decrease the levels of the Aβ monomer in the blood, resulting in a concentration gradient of Aβ from the blood to the brain. In turn, this concentration gradient promotes an increase in Aβ efflux from the brain to the peripheral blood^29^. Our result showed that the Aβ aggregates and plaque loads were significantly reduced, whilst the level of soluble Aβ was increased, in the brains of immunized mice compared to the control group. This might be due to the passage of induced serum Aβ antibodies through the BBB to bind to amyloid plaques in the brain, which are then depolymerized into the soluble Aβ form. Several studies have demonstrated the intracellular Aβ-driven effects on neuronal firing might be one of the earliest detectable triggers of AD pathology preceding and predisposing to synaptic deficits^41^,^42^,^43^. Therefore, an increase in soluble Aβ may be caused by the efflux of intracellular accumulated Aβ. Furthermore, the Aβ42 concentration in the serum of the control group declined slowly with age but this phenomenon was not observed in PP-3copy-Aβ1-6-loop123-treated mice. This might be because the “peripheral sink” pathway, by which induced serum Aβ antibodies can clear the Aβ monomers in the blood and stimulate the flow of Aβ from the brain to the peripheral blood through the BBB, results in an unchanged level of Aβ42 in the blood finally resulting in Aβ42 clearance from the brain. Therefore, our results suggested that the immunization of PP-3copy-Aβ1-6-loop123 might have restored the homeostasis of Aβ *in vivo* through a direct-targeting mechanism and the peripheral sink pathway. We also tried to assess the Aβ antibody titers in the CSF of immunized mice to confirm the antibodies could cross the BBB, but it was too difficult to extract CSF from APP/PS1 transgenic mice. Therefore, our next study will use non-human primates such as Rhesus monkeys to assess the Aβ antibody titers in the CSF for the further investigation of this novel AD immunotherapy.

For more than 2 decades, Aβ plaques have been considered the key pathogenic substances in AD pathogenesis. However, recent studies have indicated that correlations between the plaque density and severity of dementia in AD were poor^40^,^44^. Many studies have suggested that soluble Aβ oligomers may be the main cause of synaptic dysfunction and memory loss in AD, whereas the plaques might be a form of compensatory deposit^45^,^46^. Moreover, some studies suggested that the altered dynamic equilibrium between Aβ fibrils-oligomers-monomers maybe the trigger for AD^47^. This might explain why therapy targeting Aβ plaques has failed. In our study, we aimed to not only reduce amyloid deposition or dissolve Aβ plaques but also to restore Aβ homeostasis in both the serum and brain of treated mice by using active Aβ immunotherapy. Our results showed that immunization with PP-3copy-Aβ1-6-loop123 successfully reduced amyloid deposition, rescued memory loss, and restored the homeostasis of Aβ *in vivo*. Furthermore, the Aβ antibody induced by PP-3copy-Aβ1-6-loop123 reacted with soluble Aβ oligomer, and effectively inhibited Aβ aggregation and neutralized toxicity induced by Aβ oligomers.

Aβ has traditionally been characterized as a functionless catabolic byproduct, with pathogenic pathway antimicrobial activity. However, recently, it was demonstrated that Aβ is physiologically released during neuronal activity and is required for synaptic plasticity and memory in healthy subjects^48^,^49^,^50^,^51^,^52^,^53^,^54^. This must be taken into account and further investigations are needed before the use of anti-Aβ therapy is extended to healthy humans to prevent the onset on AD.

The debate about Aβ immunotherapy is ongoing. Although many clinical trials targeting Aβ have failed, the success of BIIB037 in current studied provides strong support for the ongoing use of Aβ as a therapeutic target. Recently studied proposed the combined immunization of Aβ and Tau vaccines might be a promising immunotherapy approach, and our research has laid the foundation for this new treatment strategy^10^.

## Methods

### Expression constructs

The NoV P domain (Hu/GII.4 GenBank: DQ078814.2) cDNA was synthesized by Generay Biotechnology Corporation (China) and inserted into the pET28a (+) vector to produce wild type P particles (PP). Then wild type P particle peptide sequences were used as the template to produce the PP-3copy-Aβ1-6-loop123 recombinant protein by inserting three copies of Aβ 1-6 (GGGDAEFRHGGGDAEFRHGGGDAEFRHGGG) into the sites between the G274 and T275 residues of loop 1, the S372 and N373 residues of loop 2, and the G392 and S393 residues of loop 3 via a GGG linker, respectively.

### Protein expression and purification

The wild type P particle and PP-3copy-Aβ1-6-loop123 protein vaccine were expressed in *Escherichia coli* strain BL21 (ED3) and purified by Ni-NTA affinity chromatography as reported previously^30^. The protein fractions eluted with 300 mM imidazole were used for further analyze. The eluate with the chimeric PP was loaded onto a Superdex™ 200 (GE Healthcare Life Sciences, CHA) size exclusion column to separate the 24 polymers.

### SDS-PAGE, native-PAGE, Western blot, and TEM

The molecular weights (MW) of the proteins were estimated by 13.5% SDS-PAGE. Native-PAGE was used to identify the 24-mer form of the protein: the native-PAGE gels did not contain 10% SDS and the samples were not treated with β-mercaptoethanol prior to separation.

For Western blots, the proteins were transferred onto nitrocellulose membranes (Whatman, Kent, UK) after separated by 13.5% SDS-PAGE. An anti-His tag monoclonal antibody (Invitrogen, USA) was used to analysis the particles.

The morphological characteristics of the proteins were observed using a TEM (H-7650, Hitachi, Japan). Observations were conducted by TEM (H-7650, Hitachi, Japan) with an accelerating voltage of 80 kV, and images (50 k magnification) were obtained with a CCD camera system.

### Dose response study in C57BL/6 mice

Forty-two 8-week-old female C57BL/6 mice were used to determine the optimal immunogenic dose of PP-3copy-Aβ1-6-loop123. The mice were assigned to seven groups. Three groups of seven animals were each immunized with different doses of PP-3copy-Aβ1–6-loop123 (12.5, 25, and 50 μg). A fourth group was immunized with 100 μg Aβ1-42 peptides with Freund’s adjuvant and a fifth group was immunized with 25 μg PP-3copy-Aβ1–6-loop123 with CpG as an adjuvant (TGTCGTCGTCGTTTGTCGTTTGTCGTT, synthesized by Generay Biotechnology Corporation (China)). The final two groups were immunized with PBS and CpG as blank controls. Each group was immunized four times in total at 2-week intervals by subcutaneous injection; all animals were bled via the tail vein before the immunization. The animals were sacrificed after the fourth immunization and the spleen cells were obtained for an Elispot assay. All animal studies were conducted in accordance with legal and institutional guidelines. The procedures were approved by the Ethical Committee of Care and Use of Laboratory Animals at Jilin University.

### APPswe/PS1dE9 transgenic mice and vaccine immunization

The transgenic mice used in this study, harboring a mutant presenilin 1 (PS1 A246E) and a mutant amyloid precursor protein (APPswe) gene, were provided by the Model Animal Research Center of Nanjing University. Male APPswe/PS1 transgenic mice (N = 180) were separated into three equal age cohorts (4, 6, and 9 months), presenting different stages of the disease course of AD: before the onset (4mo), directly after the onset (6mo), and long after the onset (9mo), respectively. Every cohort was divided into four groups, which were immunized with the following: PBS (n = 15), 25 μg PP (n = 15), 25 μg PP-3copy-Aβ1–6-loop123 with CpG (n = 15) and 100 μg Aβ42 peptides with Freund’s adjuvant (n = 15), respectively. Every group underwent the same immunization process: the animals were immunized four times at weeks 0, 2, 4, and 6 and then boosted at week 12. Bleeding via the tail vein was conducted every two weeks and behavior was tested at week 18–20.

### Determination of Aβ antibody titers induced by immunization

Serum Aβ-specific antibody titers induced by immunization were measured by a standard ELISA using 96-well plates coated with the Aβ1–42 peptide (100 ng/well). Serum dilutions from 1:800 to 1:51200 were used. The mouse monoclonal Aβ1–16-specific antibody 6E10 (1 mg/ml) was used for the calibration curve. Plates were read at 450 nm using a microplate reader and absorbance values over twofold greater than the background values were considered positive.

### Cytokine determination by Elispot assay (T-cell activation)

Spleen cells were prepared 15 d after the fourth dose of vaccine and were seeded at 1 × 10^6^ cells per well in 100 μL of medium. Cytokine release from plated cells was stimulated *in vitro* using 100 ng/mL Aβ1-42, as described previously^30^. Positive spots were quantified using a mouse IFN-γ Elispot kit (BD Biosciences, USA) according to the manufacturer’s recommendations.

### Aβ oligomer preparation, and the Aβ toxicity inhibition assay

The details of these assays have been described previously^30^. The Aβ antibodies were purified by saturated ammonium sulphate (SAS) precipitation from the antiserums of the immunized mice. In brief, antiserums were dissolved in normal saline and saturated ammonium sulphate was added to the mixture drop by drop. After centrifuging at 10,000 rpm for 20 min, the supernatants were removed. Normal saline was used to suspend the precipitate, which was repeated twice and then dialyzed in normal saline for 24 h. The purified antibody was stored at −80 °C.

Hexafluoro-2-propanol (HFIP) was used to prepare the Aβ oligomers^55^,^56^,^57^,^58^. Briefly, Aβ1–42 peptide was dissolved in cold hexafluoro-2-propanol (HFIP). HFIP was removed by evaporation and Aβ1–42 was dissolved in anhydrous dimethyl sulfoxide (DMSO). Then ice-cold F12 medium was added to the mixture. This solution was incubated at 4 °C for 24 h and then centrifuged at 14,000 × g for 10 min in order to remove the insoluble material. A supernatant containing oligomers was obtained.

For MTT assay, single pheochromocytoma (PC12) cells (5 × 10^4^) were seeded in 96-well plates in 100 μL/well and cultured overnight. The prepared oligomer (1 mg/ml) and different dilutions (0.05 μM and 0.1 μM) of Aβ antibodies were then added into the cell culture medium. After incubated in 37 °C for 48 h, MTT (5 mg/mL in 20 μL) was added to each well and incubated for another 4 h at 37 °C. Centrifuged the plates, and removed the supernatants carefully. DMSO (150 μL) was added to each well of the plates. Absorbance values of each well were read at 490 nm using an ELISA reader (Bio-Rad, USA).

### Inhibition of Aβ aggregation

The Thioflavin T (ThT) fluorescence protocol was used to detect the *in vitro* function of the purified antibody. Briefly, 1.6 μL THT solution (5 mM) was mixed with 3 μL Aβ stock solution (1 mg/ml in 10 mM NaOH), with or without purified antibody, into white opaque 96-well plates and aggregation was allowed to take place. The final volume was made up to 150 μL with a buffer containing 10 mM PB, 500 mM NaCl, pH 7.0. The fluorescence was subsequently measured every hour using a fluorescence microplate reader (Fluoroskan ascent FL, Thermo, USA) at an excitation wavelength of 425 nm and an emission wavelength of 460 nm.

### Morris water maze

The water maze test was performed in a white iron pool with a fixed white circular platform hidden 1–2 cm below the surface of the water (22 ± 1 °C). The acquisition phase was carried out during six consecutive days with four daily trials. Each trial lasted 60 s or until the mouse found the hidden platform; mice not finding the platform within 60 s were guided to it by the experimenter. The escape latency was recorded by an automated video tracking system (San Diego Instruments, USA). On day 7, the platform was removed from the pool and the mouse allowed to explore the pool for 60 s. The number of crossings over the target platform (where the platform was located during hidden platform training), the time spent in the target quadrant were measured.

### Nest-building test

Nest building was used to detect hippocampus damage^33^. APPswe/PS1 mice were housed in single cages containing sawdust for one week. Before testing, two pieces of compressed cotton, measuring 5 cm × 5 cm, were introduced inside the cage for nesting. The presence and quality of the nest were rated one day later on a scale from 1 to 4 as follows: score 1, not noticeably touched; score 2, partially torn up; score 3, mostly shredded but flat; score 4, perfect or nearly perfect.

### Immunohistochemistry

After mice were sacrificed, half brains were excised, fixed in 4% paraformaldehyde, and then embedded in paraffin. Longitudinal sections across the hippocampus were processed by standard procedures. After blocking with 5% normal goat serum, the sections was incubated with an anti-Aβ1-16 monoclonal antibody 6E10 (1:200, Covance, USA) at 4 °C overnight. The sections were then washed with PBS, incubated with horseradish peroxidase (HRP)-labeled sheep anti-mouse secondary antibody and then reacted with the chromagen diaminobenzidine (DAB).

### Determination of Aβ in brain and serum

Commercial ELISA kits (Uscn Life Science Inc., China) were used according to the manufacturer’s instructions to measure the concentrations of human Aβ42 and human Aβ40 in the serum and brains of the mice treated directly after the onset of AD. Human Aβ levels in the brain were also measured using commercial ELISA kits (Invitrogen, CA) according to the manufacturer’s instructions.

### Determination of the inflammatory cytokine levels in the brain of AD mouse model

The brain samples form directly after the onset cohort were homogenized in a buffer of 20 mM Tris, pH 8.5, containing complete inhibitory mixture (Roche Diagnostics, Germany), followed by ultrasonication for 1 min. After centrifugation, the supernatant was taken for measurement of cytokine levels, closely following the manufacturer’s instructions. The levels of the cytokines IL-2, IL-4, IL-6, IL-10, IL-17, IL-1β, TNF-α, and IFN-γ in the brains of AD mice following immunization were determined using commercial ELISA kits (Biolegend, USA). The WT mice of 11 month were used as control.

### Statistical analysis

Statistical analysis was performed using SPSS software (version 10.0, Chicago, IL, USA). Image-Pro Plus 6.0 software (Media Cybernetics, USA) was used for counting plaques and for the analysis of plaque areas. The results are expressed as mean values ± SEM. Statistical comparisons between groups were determined by ANOVA analyses using the S-N-K method for testing the significance of values. Each experiment was performed in triplicate, and a *p* value < 0.05 was considered statistically significant.

## Additional Information

**How to cite this article**: Fu, L. *et al*. Norovirus P particle-based active Aβ immunotherapy elicits sufficient immunogenicity and improves cognitive capacity in a mouse model of Alzheimer’s disease. *Sci. Rep.* **7**, 41041; doi: 10.1038/srep41041 (2017).

**Publisher's note:** Springer Nature remains neutral with regard to jurisdictional claims in published maps and institutional affiliations.

## Supplementary Material

Supplementary Information

## Figures and Tables

**Table 1:** The comparison of plaques characteristics in different cohorts of APP/PS1 transgenic mice after the PP-3copy-Aβ1-6-loop123 treatment.

Group	Treatment age (month)	Plaque number in hippocampus (%)	Plaque number in cerebral cortex (%)	Plaque area in hippocampus (%)	Plaque area in cerebral cortex (%)
Before the onset of AD	4–9	−48.73	−49.10	−55.53	−37.32
Directly after the onset of AD	6–11	−65.52	−46.6	−70.31	−67.93
Long after the onset of AD	9–14	−23.91	−26.80	−41.05	−14.63
