# Supplementary material for: Norovirus P particle-based active Aβ immunotherapy elicits sufficient immunogenicity and improves cognitive capacity in a mouse model of Alzheimer’s disease
Source: Sci Rep. 2017 Jan 20;7:41041. doi: 10.1038/srep41041 (PMC5247735; doi:10.1038/srep41041)
Supplement: Supplementary Information [file srep41041-s1.doc]

**Norovirus P particle-based active Aβ immunotherapy elicits sufficient immunogenicity and improves cognitive capacity in a mouse model of Alzheimer’s disease**

Lu Fu^1, 2^, Yingnan Li^1^, Yue Hu^1^, Yayuan Zheng^1^, Bin Yu^1, 2^, Haihong Zhang^1, 2^, Jiaxin Wu^1, 2^, Hui Wu^1, 2,*^ ,Xianghui Yu^1, 2*^, Wei Kong^1, 2,*^


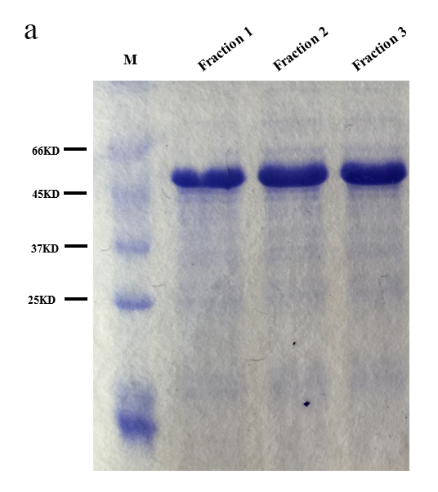

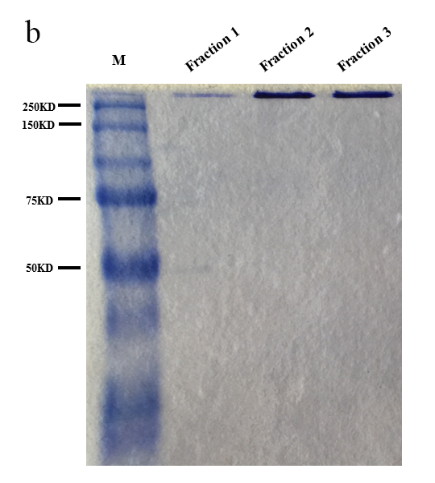

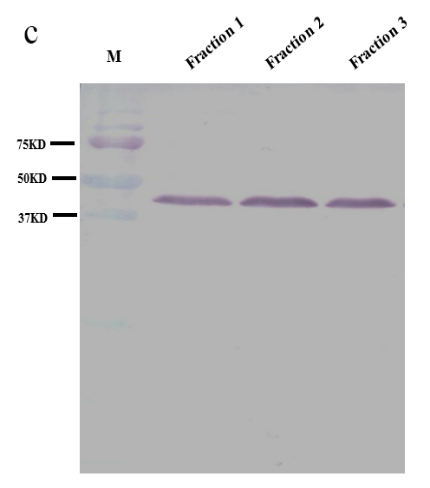


**Figure S1.**

SDS-PAGE, native-PAGE, anti-His western blot analysis of recombinant P particles in peak 1 eluted from Superdex 200 column. (a) SDS-PAGE analysis; (b) Native-PAGE analysis; (c) Anti-His western blot analysis.


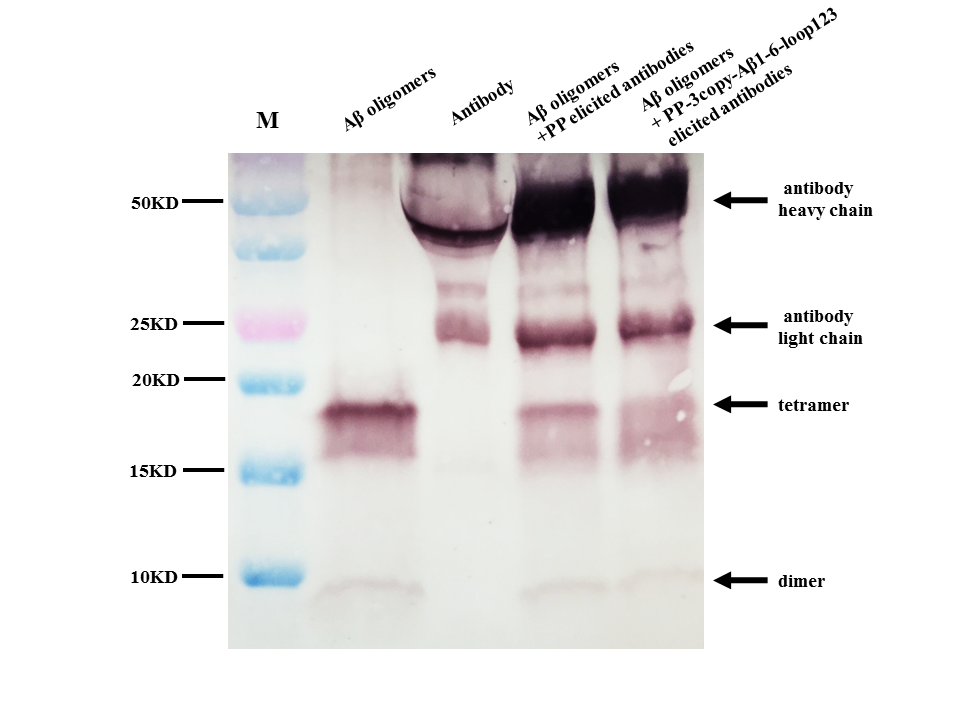


**Figure S2.**

Western blot analysis of inhibition effect of Aβ oligomers by purified Aβ42 antibodies induced by PP-3copy-Aβ1-6-loop123 treatment. Anti-Aβ1-16 monoclonal antibody 6E10 (Covance, USA) were used as the primary antibody to characterize the content of the oligomers.


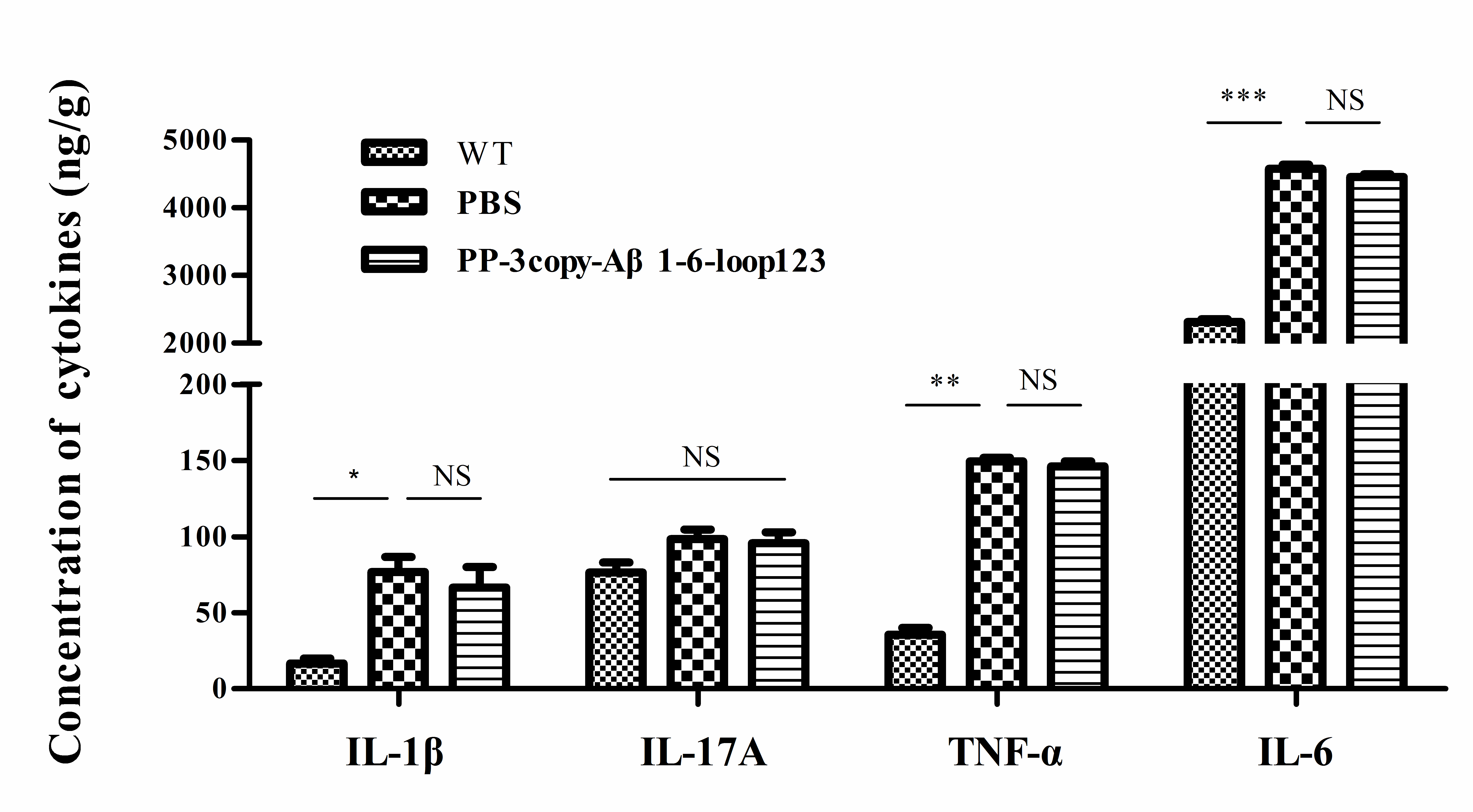


**Figure S3.**

Levels of proinflammatory cytokines in the brain of PBS or PP-3copy-Aβ1-6-loop123 immunized transgenic mice. WT mice of the same age were used as control.
